# Domestic

**Published:** 1842-01-01

**Authors:** 


### DOMESTIC.

*On the condition of the Nails in Fractured Limbs.—Hairs found under the Nail of the Thumb.*—The New York Medical Gazette for November 24th contains the following notice of an article in the last number of the British and Foreign Medical Review.

"Dr. Pitschaft mentions, as a remarkable fact, that the nails do not grow during the union of a fracture, and that the commencement of growth in them is proof that the fracture has united. Dr. Lesler is mentioned as the first who ever observed this. Does this arrest of growth depend merely on the fracture of the limb, or is it, as the editor of the British and Foreign Review suggests, but indirectly connected with the fracture, depending on the well known principle that the growth of the various horny tissues depends on the amount of waste to which they are exposed. The subject should be investigated."

Our estimable friend, as we shall ever hold him, of the Boston Medical and Surgical Journal, closes his remarks on the same article with the following implied request.

"Should any of our correspondents be possessed of knowledge upon this subject, or, by a series of inquiries which they may be induced to institute, convince themselves that such a law of the animal economy does really exist, they would confer a peculiar favour by communicating the result of their observations to the medical public."

We observed the statement in the British Review on its reception in this country, and, as it was deemed worthy of question and ingenious comment in that excellent journal, we thought it worthy of refutation, and accordingly requested our valuable correspondent, Dr. Edward Hartshorne, Resident Surgeon of the Pennsylvania Hospital, to observe the condition of the nails in a few cases of fracture of important bones. From the result of his observations we are inclined to believe that, in the Pennsylvania Hospital, during the year 1841, the growth of the nails has not been checked by the process of eliminating callus in fractures; but, in these days of tabular deduction, we are by no means prepared to contend against the possible existence of such a pathological law on the continent of Europe. Numerous observations, under a multitude of circumstances, are required to establish such a principle in pathology; for every variety of temperament and idiosyncracy must be regarded as among the data in the calculation; and every peculiarity in the influence of the non-naturals over individual constitutions must be estimated in order to arrive at a legitimate conclusion. Moreover, having been twice subjected to the fracture of limbs in our own proper person, we are convinced that the arrest of development in the horny tissues—if it exist as a symptom of such accidents in general—is not universally observed; for we have never experienced any deficiency of this species of literary aliment on these occasions. The statement of the British reviewer does not convince us—in the absence of all convenient opportunity of reference to the original papers of Doctors Lesler and Pitschaft—that due care was exercised in noting the peculiar habit of the patients upon whose history the conclusion has been founded. They might have been in the habit of biting their nails.

But—a truce to bantering.—The cure of a fracture is a laborious process, and as all vital operations at a distance from the seat of the injury are carried on with difficulty and diminished energy in such cases, it is possible that the secretions of cuticle, hair, and the nails, partake in the general deficiency of vital action consequent upon its concentration about the site of the injury. A neglect of this genuine "law of the animal economy," and the injurious habit of confining patients labouring under fracture to a low, instead of a generous diet, is unquestionably among the most frequent causes of pseudarthrosis. But when the attempt to make the deficient, though not arrested, growth of the cuticular appendages, supposing it to be observed in certain cases, a test of the completion of union,—treating the whole subject with proper dignity and the respect justly due to those with whom the idea originated, we unhesitatingly pronounce it sheer nonsense.

Under the same head we should class the story of the young girl at the West, who was said to have discharged certain hairs from beneath the thumb nail, and whose case has recently made much noise in the journals as a wonder, though now known to have been an innocent imposition. Had the "phenomenon" really occurred, it would have been nothing remarkable, as the dermoid tissue is every where capable of producing the secretory apparatus necessary for the formation of hair ; and any part, even of the internal mucous surfaces, if it could be kept dry and exposed to the air consistently with continued life, would be speedily transformed into skin ;—a fact well known to all physiologists. We have seen hairs growing pretty abundantly upon prolapsed and transformed mucous membrane in an old case of prolapsus ani.

